# Effect of Neuromuscular Electrical Stimulation After Total Knee Arthroplasty: A Systematic Review and Meta-Analysis of Randomized Controlled Trials

**DOI:** 10.3389/fmed.2021.779019

**Published:** 2021-12-03

**Authors:** Linbo Peng, Kexin Wang, Yi Zeng, Yuangang Wu, Haibo Si, Bin Shen

**Affiliations:** ^1^Department of Orthopedics, Orthopedic Research Institute, West China Hospital, Sichuan University, Chengdu, China; ^2^Department of Clinical Research Management, West China Hospital, Sichuan University, Chengdu, China

**Keywords:** total knee arthroplasty, neuromuscular electrical stimulation, quadriceps muscle strength, pain, function, systematic review, meta-analysis

## Abstract

**Background:** This systematic review and meta-analysis aimed to evaluate the effect of neuromuscular electrical stimulation (NMES) on quadriceps muscle strength, pain, and function outcomes following total knee arthroplasty (TKA).

**Methods:** PubMed/Medline, Embase, Web of Science, CENTRAL, Scopus, PsycINFO, PEDro, CINAHL, CNKI, and Wanfang were systematically searched for randomized controlled trials (RCTs) from their inception to 18 June 2021.

**Results:** Nine RCTs that involving 691 patients were included in the meta-analysis. Our pooled analysis showed that NMES improved quadriceps muscle strength after TKA within 1 months [standardized mean difference (SMD): 0.81; 95% CI: 0.51–1.11], 1–2 months (SMD: 0.55; 95% CI: 0.13–0.97), 3–4 months (SMD: 0.42; 95% CI: 0.18–0.66), and 12–13 months (SMD: 0.46; 95% CI: 0.18–0.74), pain between 1 and 2 months [mean difference (MD): −0.62; 95% CI: −1.04 to −0.19], pain between 3 and 6 months (MD: −0.44; 95% CI: −0.74 to −0.14) Western Ontario and McMaster Universities Osteoarthritis Index (WOMAC) between 3 and 4 months (MD: −0.43; 95% CI: −0.82 to −0.05), timed up and go test (TUG) within 1 month (MD: −2.23; 95% CI: −3.40 to −1.07), 3 minutes walk test between 3 and 6 months (MD: 28.35; 95% CI: 14.55–42.15), and SF-36 MCS between 3 and 6 months after TKA (MD: 4.20, 95% CI: 2.41–5.98).

**Conclusion:** As a supplementary treatment after TKA, postoperative NMES could improve the short-term to long-term quadriceps muscle strength, mid-term pain, and mid-term function following TKA. However, many outcomes failed to achieve statistically meaningful changes and minimal clinically important difference (MCID), thus the clinical benefits remained to be confirmed.

**Level of Evidence:** Therapeutic level I.

**Systematic Review Registration:** https://www.crd.york.ac.uk/PROSPERO/, identifier CRD42021265609.

**What is Known:** Neuromuscular electrical stimulation was regarded as a potential approach to improve muscle contractility and postoperative quadriceps weakness. With relevant studies published, the advantage of NMES on quadriceps muscle strength, pain, and function outcomes following TKA remains controversial.

**What is New:** This systematic review and meta-analysis is the first to identify that postoperative NMES could improve the quadriceps muscle strength, pain, and function following the TKA surgery. The quality of evidence ranged from good to high. However, many outcomes failed to achieve statistically meaningful changes and MCID, thus the clinical benefits remained to be confirmed.

## Introduction

Total knee arthroplasty (TKA) is one of the most common and cost-effective procedures for patients with end-stage osteoarthritis of the knee, which has been performed with increasing frequency in recent years (1, 2). Although TKA provides patients with reduced pain and a functional range of motion (ROM) of the knee joint, quadriceps strength impairment is common following the surgery (3). Besides, almost all patients suffer from postoperative pain with different levels, which affect postoperative satisfaction and outcomes (4). Studies have shown that nearly 20% of primary TKA patients were not satisfied with their outcomes following the surgery (5).

Standardized physical therapy and pharmacologic analgesia improve muscle strength and pain after TKA (6). However, the content of rehabilitation varies worldwide (7, 8). Electrical stimulation is effective in accelerating recovery from surgery (9). Neuromuscular electrical stimulation (NMES) has been utilized since the eighteenth century (10). It was regarded as a potential approach to improve muscle contractility and postoperative quadriceps weakness (11). A systematic review involving a total of 933 participants found that NMES may be an effective treatment for muscle weakness and should be regarded as a crucial part of rehabilitation programs (12). The advantages of NMES have been emphasized in many diseases such as anterior cruciate ligament injury, neck pain, stroke, and cerebral palsy (13–17).

Recently, some studies have examined the effect of NMES following TKA but remain controversial (18–23). The clinical effectiveness of NMES following TKA on quadriceps muscle strength, pain, and function outcomes remains unclear. We conduct this meta-analysis to evaluate the effect of NMES on quadriceps muscle strength, pain, and function outcomes following TKA further.

## Methods

This study was based on the previous published RCTs. Thus, the ethical approval and consent to participate were not necessary. This systematic review and meta-analysis is performed following the Cochrane Handbook for Systematic Reviews of Interventions (24) and Preferred Reporting Items for Systematic Reviews and Meta-Analyses (PRISMA) guidelines (25). The protocol was registered in the PROSPERO (Registration number: CRD42021265609).

### Search Strategy

The PubMed/Medline, Embase, Web of Science, the Cochrane Central Register of Controlled Trials (CENTRAL), Scopus, PsycINFO, Physiotherapy Evidence Database (PEDro), CINAHL, China National Knowledge Infrastructure (CNKI), and Wanfang (a Chinese database) were systematically searched for randomized controlled trials (RCTs) from their inception to 18 June 2021 by two independent reviewers (LBP and KXW). The search strategies were shown in Appendix 1.

### Eligibility Criteria

The studies included in the meta-analysis were required to meet the following inclusion criteria: (1) Patients: adult patients undergoing primary TKA; (2) Intervention: postoperative NMES. NMES was utilized in the intervention group after the TKA surgery. Patients who received preoperative NMES were excluded; (3) Comparison: conventional rehabilitation or conventional physical therapy. Patients who received any form of electrical stimulation in the control group were excluded; (4) Outcomes: The primary outcome measures, such as quadriceps muscle strength [maximal volitional isometric contraction (MVIC)], physiological cost index (PCI), pain such as visual analog scale (VAS), and numerical pain rating scale (NPRS), Western Ontario and McMaster Universities Osteoarthritis Index (WOMAC), Timed Up and Go Test (TUG), Stair-Climbing Test (SCT), 3 Minutes Walk Test (3MWT), 6 Minutes Walk Test (6MWT), range of motion (ROM), and 36-Item Short-Form Health Survey (SF-36); (5) Study design: randomized controlled trials (RCTs); language and published time restrictions were not employed.

Maximal volitional isometric contraction is a classic method to calculate muscle strength for patients with neuromuscular disorders by providing intrinsic factors such as units of kilograms and Newtons of force (26). As a measure of energy cost, PCI was calculated by dividing the heart rate increase (heart rate at the end of the 3MWT minus resting heart rate) by walking speed (m/min) (18, 21). A lower PCI score indicated a lower energy cost during walking (27). To assess the TUG score, patients were asked to rise from an armchair, walk 3 m away, then turn and walk back to sit down on the same chair (28, 29). The TUG is an excellent representation of essential mobility, strength, balance, and agility (29). The SCT was used to assess the lower extremity strength, power, and balance (29). The 3MWT is a simple, non-incremental, and easy to conduct submaximal strength test (30). As a standard walking test, 6MWT has been widely used to determine the progress following rehabilitation intervention (31). The SF-36 is the most widely used health-related quality-of-life (HRQoL) in the USA (32). The SF-36 is consists of eight individual subscales. Scores of those subscales can be combined into two higher-order summary scores: PCS and MCS (33).

### Study Selection

Firstly, all the identified studies were imported into the Endnote X9 (Thomson Reuters, CA, USA). After removing the duplicate studies, two reviewers (YGW and HBS) scanned the titles, abstracts, and full texts independently. Any disagreements were resolved by discussion with a senior reviewer (YZ). Commentaries, letters, case reports, trial protocols, reviews, and retrospective studies were excluded from our systematic review and meta-analysis.

### Data Extraction

Two authors (LBP and YGW) extracted the following data independently and discussed with a senior reviewer (HBS) if disagreements existed. The extracted data including the publication data (the name of the author; publication year; country; study design), demographic characteristics [number of patients, age, sex, body mass index (BMI)], characteristics of the intervention (frequency, duration, intensity of the NMES program), rehabilitation type of the control group, outcomes data (quadriceps muscle strength, PCI, VAS, NPRS, WOMAC, TUG, SCT, 3MWT, 6MWT, ROM, and SF-36).

### Study Quality Assessment

Two authors (KXW and YZ) evaluated the methodological quality of the included studies independently with the Cochrane bias risk assessment tool and discussed with a senior reviewer (BS) if any disagreements existed (34). Each study was documented with low, high, or unclear risk of bias in each domain.

### Statistical Analysis

The review manager software (RevMan 5.3, Oxford, United Kingdom) was used to conduct our meta-analysis and produce forest plots. All the continuous variable outcomes were presented as the mean difference (MD) with a 95% CI to calculate the total effect of NMES on patients following TKA. The standardized mean difference (SMD) was used to calculate the total effect if different scales were utilized among the studies (35). *I*^2^ statistics measured heterogeneity among the studies. The random-effects model was used if substantial heterogeneity exists (*P* < 0.05 or *I*^2^ > 50%). If not, the fixed-effects model was adopted. A *P* < 0.05 demonstrated a statistically significant difference.

## Results

### Study Selection

**Figure 1:**
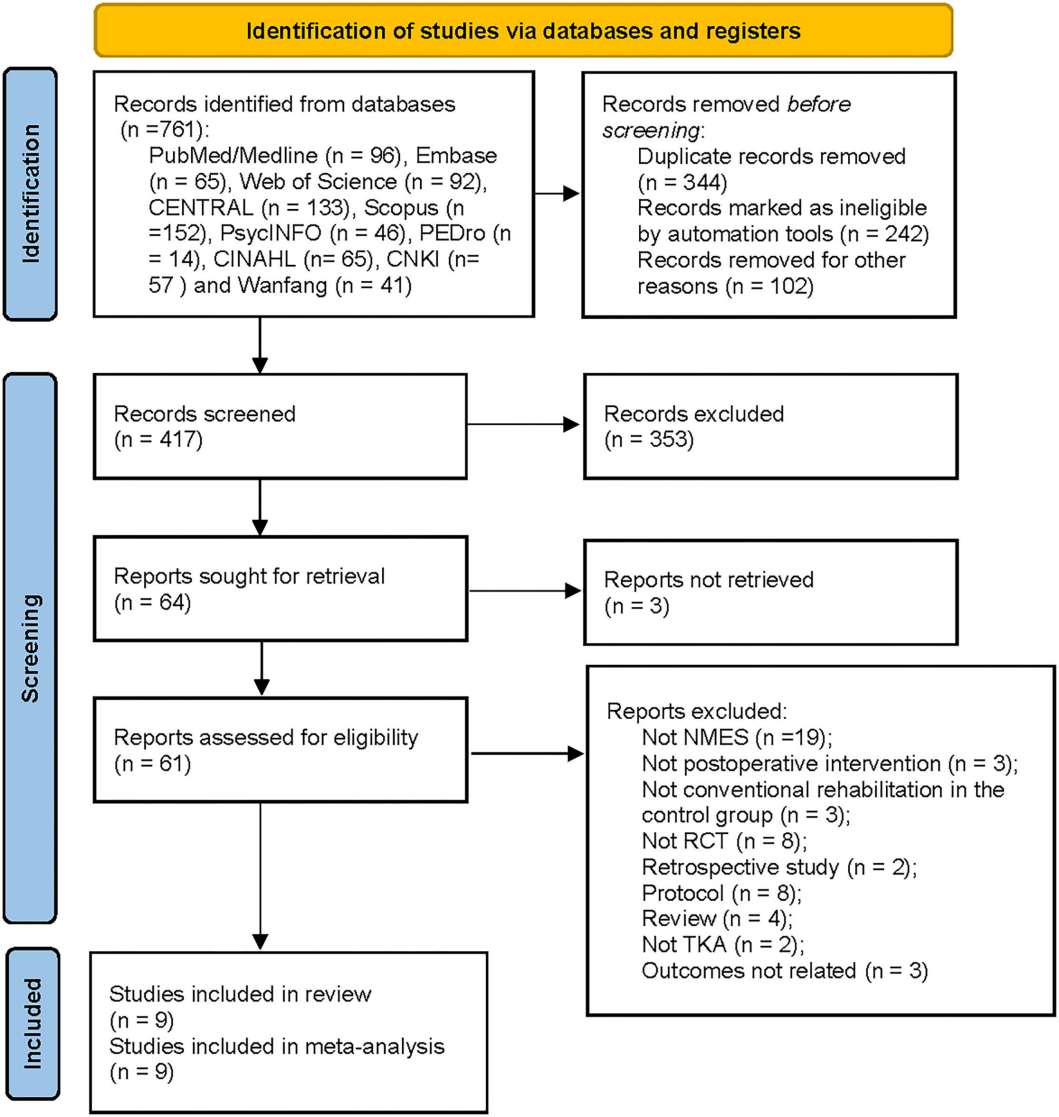
Flow chart of studies selection according to preferred reporting items for systematic reviews and meta-analyses guidelines. EMBASE, Excerpta Medica Database; CENTRAL, Cochrane Central Register of Controlled Trials; PEDro, Physiotherapy Evidence Database; CNKI, China National Knowledge Infrastructure; TKA, Total Knee Arthroplasty; NMES, Neuromuscular electrical stimulation; RCT, Randomized Controlled Trial.

A total of 761 studies were identified from the initial search. After removing 344 records for duplicates, 353 studies were excluded by screening the title and abstract. After excluding three studies for not being retrieved, the remaining 61 studies were screened the full-text for eligibility. Fifty-two reports were excluded by screening the full-text: not NMES (*n* = 19); not postoperative intervention (*n* = 3); not conventional rehabilitation in the control group (*n* = 3); not RCT (*n* = 8); retrospective study (*n* = 2); protocol (*n* = 8); review (*n* = 4); not TKA (*n* = 2); and outcomes not related (*n* = 3). The remaining nine RCTs involving 691 patients met the eligibility criteria and were included in the meta-analysis (18, 19, 21, 22, 36–40). The PRISMA flow diagram was shown in Figure 1.

### Study Characteristics

Among all the included studies, eight were 2-arm RCTs (18, 19, 21, 22, 36–38, 40) and one was 3-arm RCTs (39). The average sample size was 77 patients (ranging from 30 to 200). The follow-up periods of each article ranged from 4 to 52 weeks. Three hundred and fifty-seven patients received NMES therapy following TKA surgery, while 334 received conventional rehabilitation therapy. The included trials were performed in different countries: one in the UK (18), four in the USA (19, 22, 37, 40), one in Spain (36), one in Greece (21), one in Turkey (38), and one in Japan (39). All the characteristics of included studies were presented in Table 1.

**Table 1:** Characteristics of studies included in the meta-analysis.

**Author(s)/year/country/design**	**Patients (I: C); female proportion (I: C)**	**Age (I: C)**	**BMI (I: C)**	**Intervention vs. control**	**Intervention frequency, duration, and time**	**Intervention intensity**	**Main outcome measures**
Avramidis et al. /2003/UK/2-arm RCT	15(I):15(C); 10/15(I):12/15(C)	68.20 ± 10.59(I): 71.20 ± 7.83(C)	Not mentioned	NMES+ conventional PT vs. Conventional PT	NMES (40 Hz, 300 μs) of the vastus medialis for 2 h on each occasion, twice daily, from the second day postoperative to the 6 weeks postoperative	Maximum tolerable intensity	3MWT, PCI, HSS at 6, 12 weeks postoperatively
Petterson et al./2009/US/2-arm RCT	100(I): 100(C); 47/100(I): 45/100(C)	65.3 ± 8.3(I): 65.2 ± 8.5(C)	29.67 ± 4.85(I): 29.99 ± 3.90(C)	NMES+ exercise vs. Exercise	NMES 2 or 3 times per week for 6 weeks with a minimum requirement of 12 therapy visits	Maximum tolerable intensity	SF-36 (PCS, MCS), KOS-ADLS, pain-KOS, TUG, SCT, 6MWT, active flexion ROM, active extension ROM and CAR (NMVIC, newtons/BMI) at 3, 12 months postoperatively
Valdés et al./2010/Spain/2-arm RCT	39(I): 44(C); 25/39(I): 25/44(C)	72 ± 6(I): 70 ± 7(C)	32.3 ± 4.7(I): 32.4 ± 6.3(C)	NMES+ standard rehabilitation vs. Standard rehabilitation	NMES (65 Hz, 300 μs, 15–30 mA) of feedback to the quadriceps for 15 min once a day from the day after surgery	Not mentioned	BA, TUG, WOMAC pain, WOMAC stiffness, WOMAC function at 1, 3 months; LOS
Avramidis et al. /2011/Greece/2-arm RCT	35(I): 35(C); 28/35(I): 29/35(C)	70.54 ± 4.68(I): 70.66 ± 3.73(C)	27.38 ± 2.65(I): 27.14 ± 3.31(C)	NMES+ conventional physiotherapy vs. Conventional physiotherapy	NMES (40 Hz, 300 μs) of the vastus medialis muscle twice daily for 2 h from the second postoperative day	Maximum tolerable intensity	AKSS, OKS, SF-36, 3MWT, PCI at 6, 12, and 52 weeks postoperatively
Stevens-Lapsley et al./2012/US/2-arm RCT	35(I): 31(C); 20/35(I): 16/31(C)	66.2 ± 9.1(I): 64.8 ± 7.7(C)	27.1 ± 4.9(I): 31.2 ± 4.2(C)	NMES+ standard rehabilitation vs. Standard rehabilitation	NMES (600 μs) twice daily from 2 days after surgery	Maximum tolerable intensity	Quadriceps and hamstring muscle strength, TUG, SCT, 6MWT, NPRS, active flexion ROM, active extension ROM, SF-36 (PCS, MCS), WOMAC, GRS at 3.5, 6.5, 13, 26, 52 weeks postoperatively
Levine et al./2013/US/2-arm RCT	35(I): 35(C); 25/35(I): 21/35(C)	68.1(I): 65.1(C)	30.6(I): 31.9(C)	NMES+ ROM exercise vs. therapist-managed PT	NMES used from the second day postoperatively	Not mentioned	KSS pain, KSS function, WOMAC, passive flexion ROM, passive extension ROM, TUG at 6 weeks and 6 months postoperatively
Demet et al./2015/Turkey/2-arm RCT	30(I): 30(C); 28/30(I): 29/30(C)	66.2 ± 7.2(I): 64.6 ± 6.6(C)	29.1 ± 3.9(I): 30.1 ± 4.6(C)	NMES+ exercise vs. Exercise	NMES (30–100 Hz, 400 μs, 28–90 mA) of the vastus medialis muscle for 30 min, 5 days a week from the first day postoperatively.	Maximum tolerable intensity	flexion ROM, extension ROM, TUG, WOMAC, SF-36, VAS at 1, 3 months postoperatively
Yoshida et al./2017/Japan/3-arm RCT	22 (sNMES): 22 (mNMES): 22 (Control); 18/22 (sNMES): 18/22 (mNMES): 20/22 (Control)	71.6 ± 7.0 (sNMES):75.9 ± 4.7 (mNMES):72.5 ± 6.2 (Control)	25.4 ± 2.2 (sNMES): 24.6 ± 2.9 (mNMES): 25.8 ± 3.3 (Control)	sNMES+ standard rehabilitation vs. mNMES+ standard rehabilitation vs. Standard rehabilitation	sNMES (100 Hz, 1 ms, 10–15 mA, 45 min/day) and mNMES (100 Hz, 1 ms, 15–38 mA, 45 min/day) 5 days/week for 2 weeks from the second weeks postoperatively	Sensory-level intensity (sNMES) and maximum tolerable intensity (mNMES)	MVIC, LSMM, TUG, 2MWT, VAS (0–100 mm), passive flexion ROM and passive extension ROM at 2 weeks postoperatively
Klika et al./2020/US/2-arm RCT	24(I): 22(C); 18/24(I): 17/22(C)	65 ± 5.8(I): 65 ± 7.6(C)	Not mentioned	NMES+ standard PT vs. Standard PT	NMES (15–85 V, 50 pps, 5 ms) for 200 min/week, 12 weeks from the day of surgery	Maximum tolerable intensity	Quadriceps strength, ROM, resting pain, TUG, SCT, KOOS and VR-12 at 3, 6, and 12 weeks postoperatively

*I, Intervention; C, Control; BMI, body mass index; RCT, randomized controlled trial; NMES, neuromuscular electrical stimulation; PT, physical therapy; 3MWT, 3-minute walking test; PCI, hysiological Cost Index; HSS, Hospital for Special Surgery knee score; ROM, range of motion; TUG, timed up and go test; 6MWT, 6-minute walk test; MVIC, normalized maximal volitional isometric contraction; CAR, the central activation ratio; BMI, body mass index; SCT, stair climbing test; SF-36, Short Form 36; PCS, physical component score; MCS, mental component score; KOS ADLS, Knee Outcome Survey Activities of Daily Living scale; BA, balance articular; WOMAC, Western Ontario and McMaster Universities Osteoarthritis Index; LOS, length of stay; AKSS, American Knee Society clinical score; OKS, Oxford knee score; NPRS, Numeric Pain Rating Scale; GRS, global rating scale; KSS, knee Society score; VAS, visual analogue scale; sNMES, sensory-level neuromuscular electrical stimulation; mNMES, motor- level neuromuscular electrical stimulation; LSMM; leg skeletal muscle mass; 2MWT, 2-minute walk test; KOOS, Knee injury and Osteoarthritis Outcome Score; VR-12, veterans rand-12*.

### Interventions

All the involved patients received similar conventional rehabilitation/physical therapy/exercise. Besides, patients in the NMES groups received similar NMES therapy in all the included nine RCTs. The frequency of NMES ranged from 30 to 100 Hz, and the duration ranged from 300 μs to 1 ms in six studies (18, 21, 22, 36, 38, 39). Klika et al. set the frequency as 50 pps and the pulse width as 5 ms (40). Two studies did not document the frequency and duration data of the NMES protocol (19, 37). Three RCTs reported that the NMES therapy was used twice daily (18, 21, 22). Petterson et al. (19) conducted NMES therapy 2 or 3 times per week in the experimental group, while the other two pieces of literature (38, 39) utilized it 5 days per week. Valdés et al. adopted the NMES for 15 min once a day began from the day after surgery (36). NMES was conducted in the research of Klika et al. for 200 min per week (40). Seven studies utilized the NMES with maximum tolerable intensity (18, 19, 21, 22, 38–40). The other two RCTs did not report the intensity data of NMES (36, 37). The intervention characteristics were shown in Table 1.

### Study Quality Assessment

**Figure 2:**
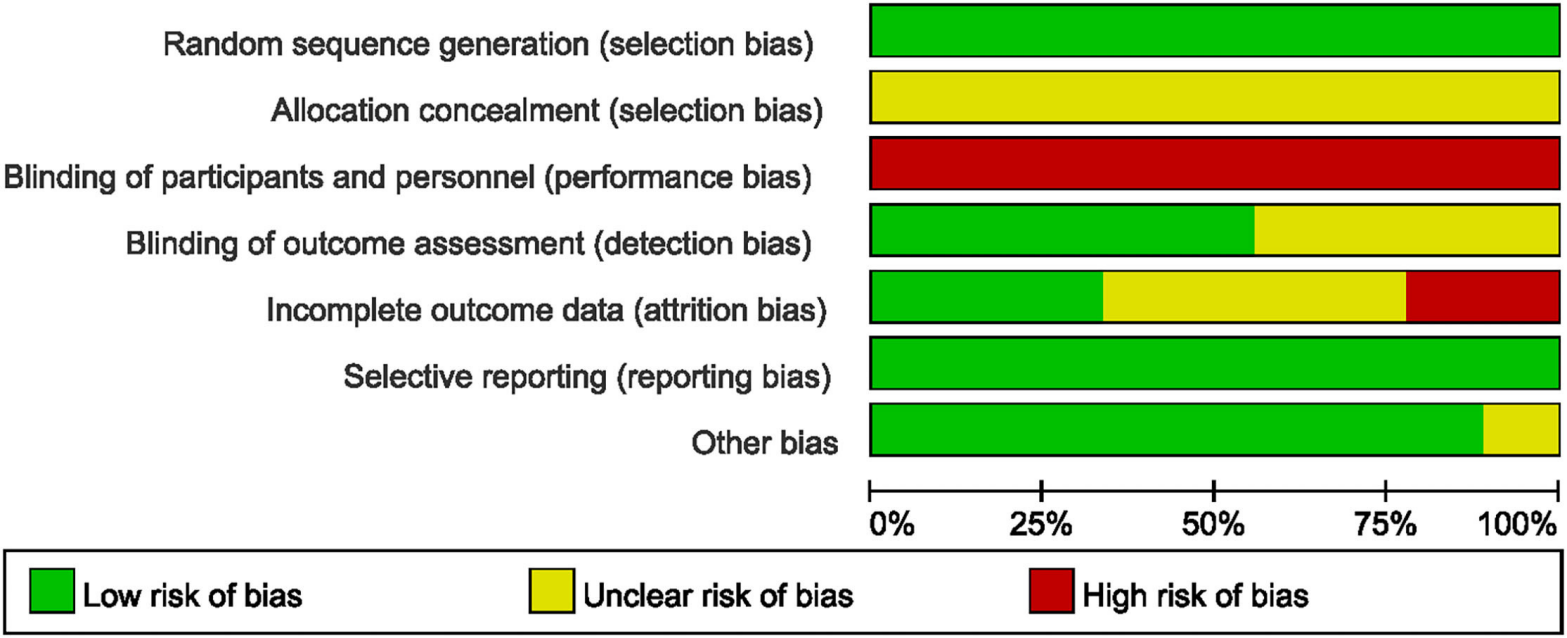
Risk of bias graph across all included studies.

**Figure 3:**
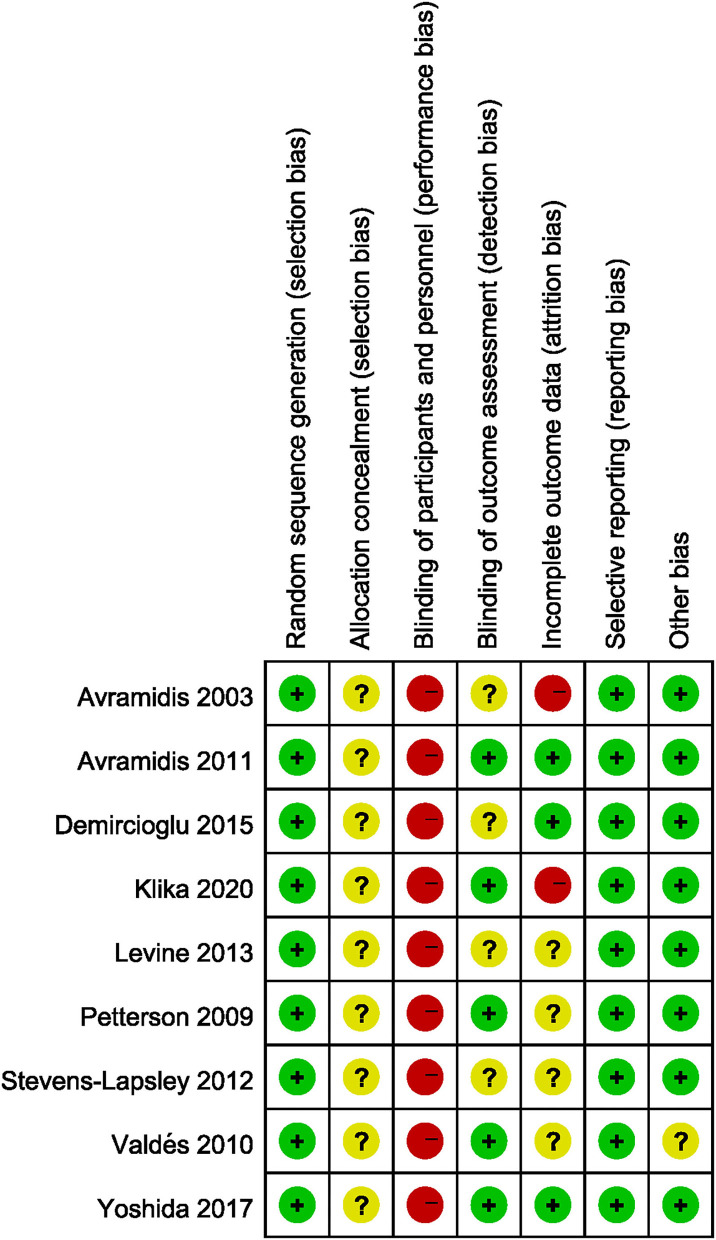
Risk of bias summary for each included studies.

The risk of bias of all the included studies varied substantially. Allocation concealment bias was unclear in all nine studies (18, 19, 21, 22, 36–40). All the studies failed to achieve performance bias (18, 19, 21, 22, 36–40). Five RCTs implied the blinded assessors in their studies (19, 21, 36, 39, 40). The risk of bias graph for each study and the risk of bias summary was shown in Figures 2, 3.

### Outcomes

#### Quadriceps Muscle Strength

**Figure 4:**
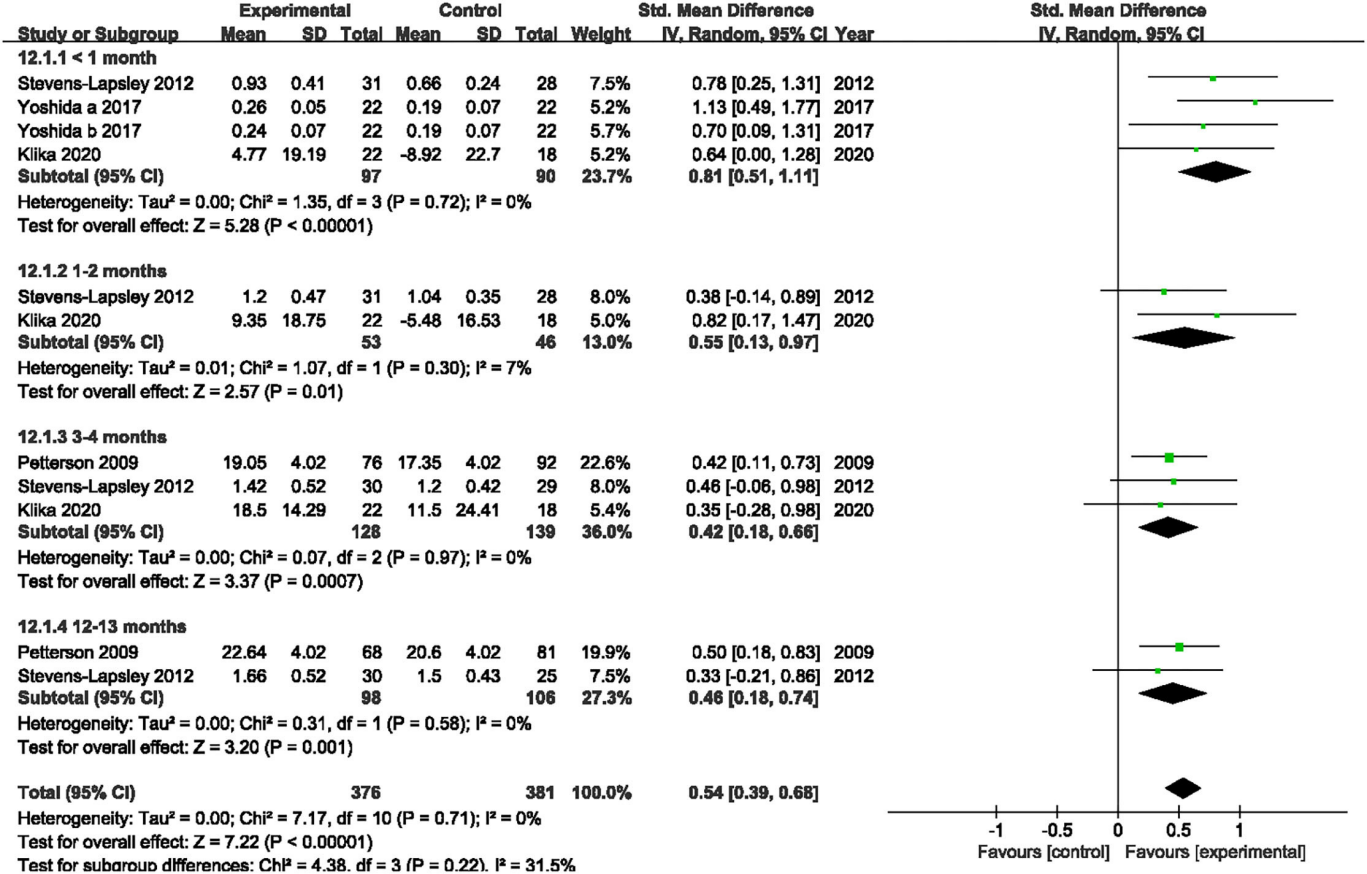
Forest plots of meta-analysis of the effect of neuromuscular electrical stimulation (NMES) vs. conventional rehabilitation on quadriceps muscle strength.

Four studies reported the quadriceps muscle strength (19, 22, 39, 40). Since the included studies adopt different intrinsic factors to normalize MVIC values, the SMD was used to calculate the total effect of quadriceps muscle strength. Our pooled analysis involving four studies indicated that NMES improved MVIC after TKA within 1 month (SMD: 0.81; 95% CI: 0.51–1.11, *P* < 0.01, *I*^2^ = 0%), 1–2 months (SMD: 0.55; 95% CI: 0.13–0.97, *P* = 0.01, *I*^2^ = 7%), 3–4 months (SMD: 0.42; 95% CI: 0.18–0.66, *P* < 0.01, *I*^2^ = 0%), and 12–13 months (SMD: 0.46; 95% CI: 0.18–0.74, *P* < 0.01, *I*^2^ = 0%). There was no significant heterogeneity (*I*^2^ = 0, 7, 0, and 0%, respectively). The forest plot of quadriceps muscle strength was shown in Figure 4.

#### PCI

**Figure 5:**
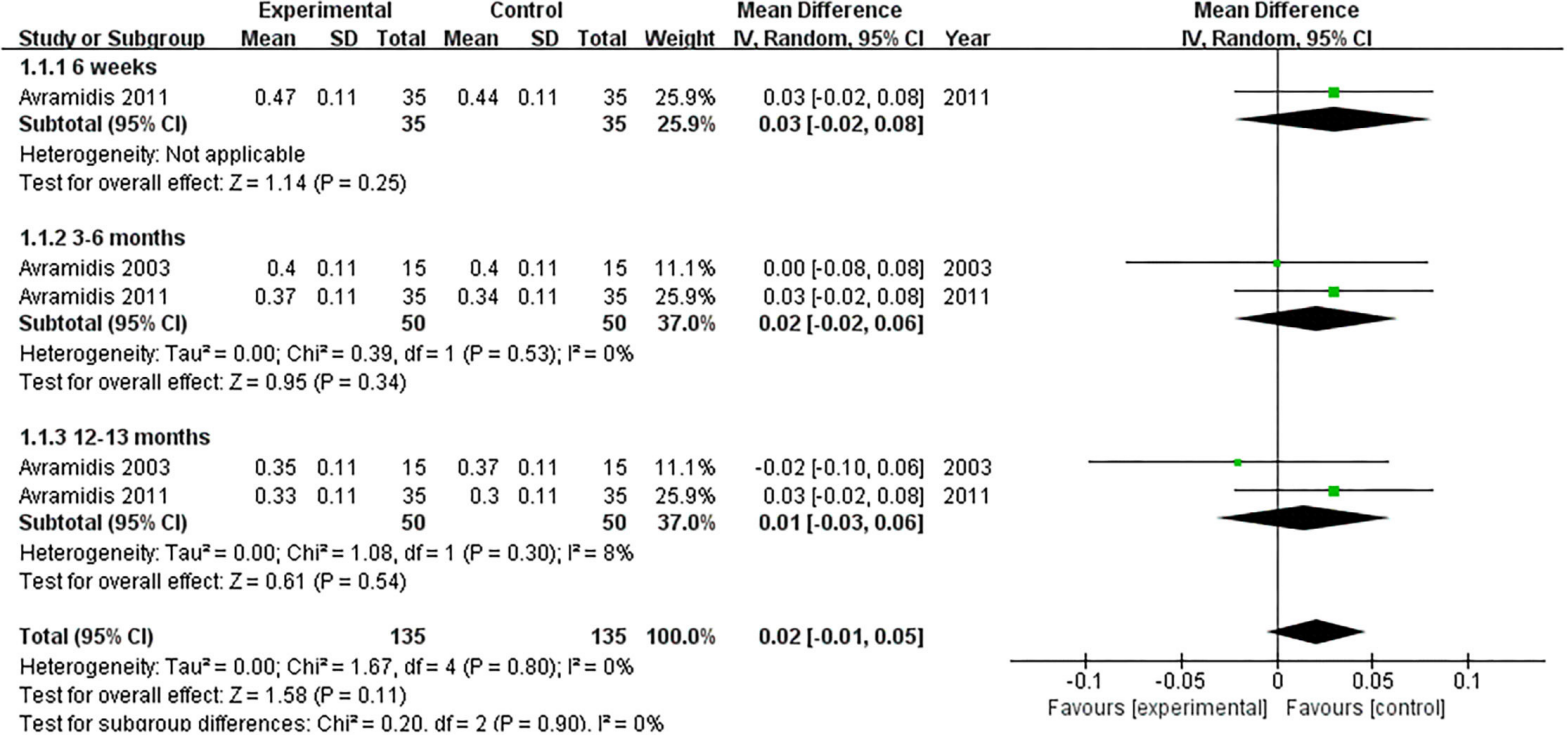
Forest plots of meta-analysis of the effect of NMES vs. conventional rehabilitation on physiological cost index.

Two studies evaluated the PCI (18, 21). NMES could not improve PCI between 3 and 6 months or 12 and 13 months after TKA compared with the control group (MD: 0.02; 95% CI: −0.02–0.06, *P* = 0.34; MD: 0.01; 95% CI: −0.03–0.06, *P* = 0.54; respectively). We found no significant heterogeneity (*I*^2^ = 0%; *I*^2^ = 8%; respectively). The forest plot of PCI was shown in Figure 5.

#### Pain

**Figure 6:**
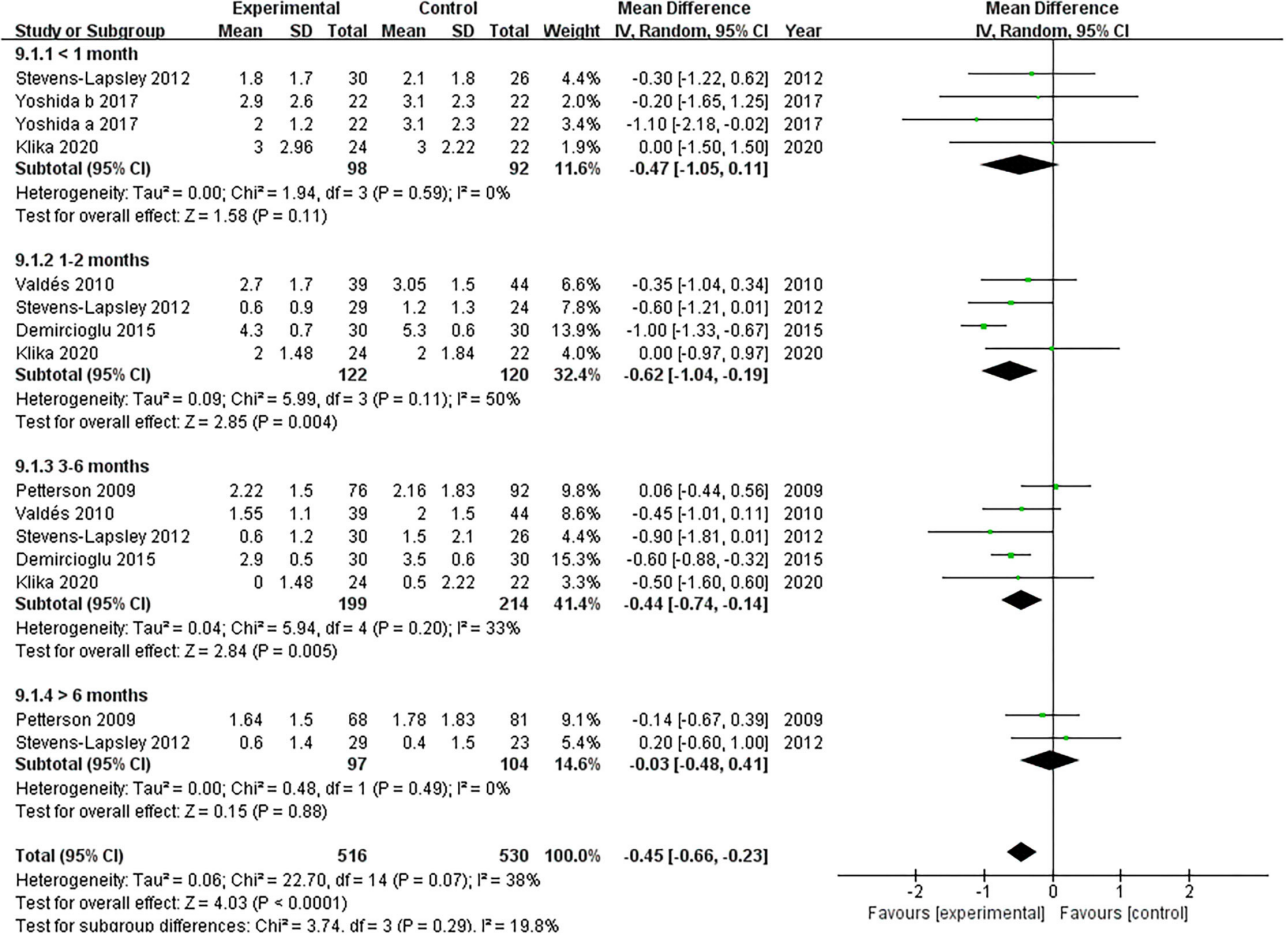
Forest plots of meta-analysis of the effect of NMES vs. conventional rehabilitation on pain.

Five studies assessed the pain scores during the rehabilitation procedure (19, 22, 38–40). Our pooled analysis comprising 168 patients demonstrated that NMES could not improve pain after TKA compared with the control group (MD: −0.47; 95% CI: −1.05–0.11, *P* = 0.11). No significant heterogeneity was found (*I*^2^ = 0%). The NMES significantly improved pain between 1 and 2 months after TKA (MD: −0.62; 95% CI: −1.04 to −0.19, *P* = 0.004). No significant heterogeneity was detected (*I*^2^ = 50%). NMES improved pain between 3 and 6 months after TKA without significant heterogeneity (MD: −0.44; 95% CI: −0.74 to −0.14, *P* = 0.005, *I*^2^ = 33%). Besides, no significant difference was found among the groups for more than 6 months (MD: −0.03; 95% CI: −0.48–0.41, *P* = 0.88, *I*^2^ = 0%). The forest plot of the pain score was shown in Figure 6.

#### WOMAC

**Figure 7:**
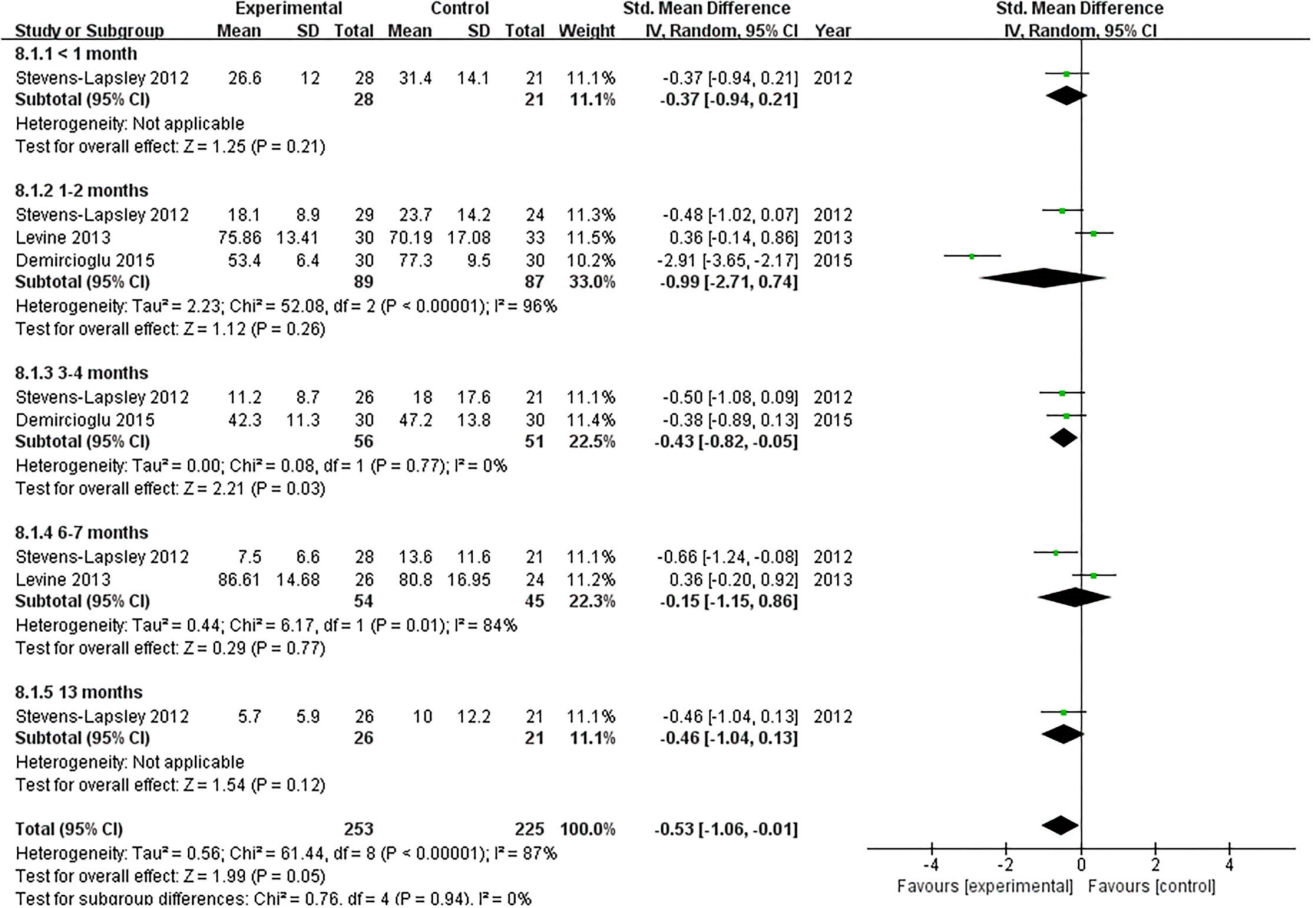
Forest plots of meta-analysis of the effect of NMES vs. conventional rehabilitation on the Western Ontario and McMaster Universities Osteoarthritis Index.

A total of three studies reported the effect of NMES on WOMAC following TKA (22, 37, 38). Our pooled analysis involving three studies (22, 37, 38) revealed that NMES could not improve WOMAC between 1 and 2 months (SMD: −0.99; 95% CI: −2.71–0.74, *P* = 0.26). There was significant heterogeneity between the articles (*I*^2^ = 96%). Nevertheless, NMES significantly improved WOMAC between 3 and 4 months after TKA (MD: −0.43; 95% CI: −0.82 to −0.05, *P* = 0.03). No significant heterogeneity was detected among the studies (*I*^2^ = 0%). We failed to find a significant difference in WOMAC between 6 and 7 months (MD: −0.15; 95% CI: −1.15–0.86, *P* = 0.77, *I*^2^ = 84%). The forest plot of WOMAC was shown in Figure 7.

#### TUG

**Figure 8:**
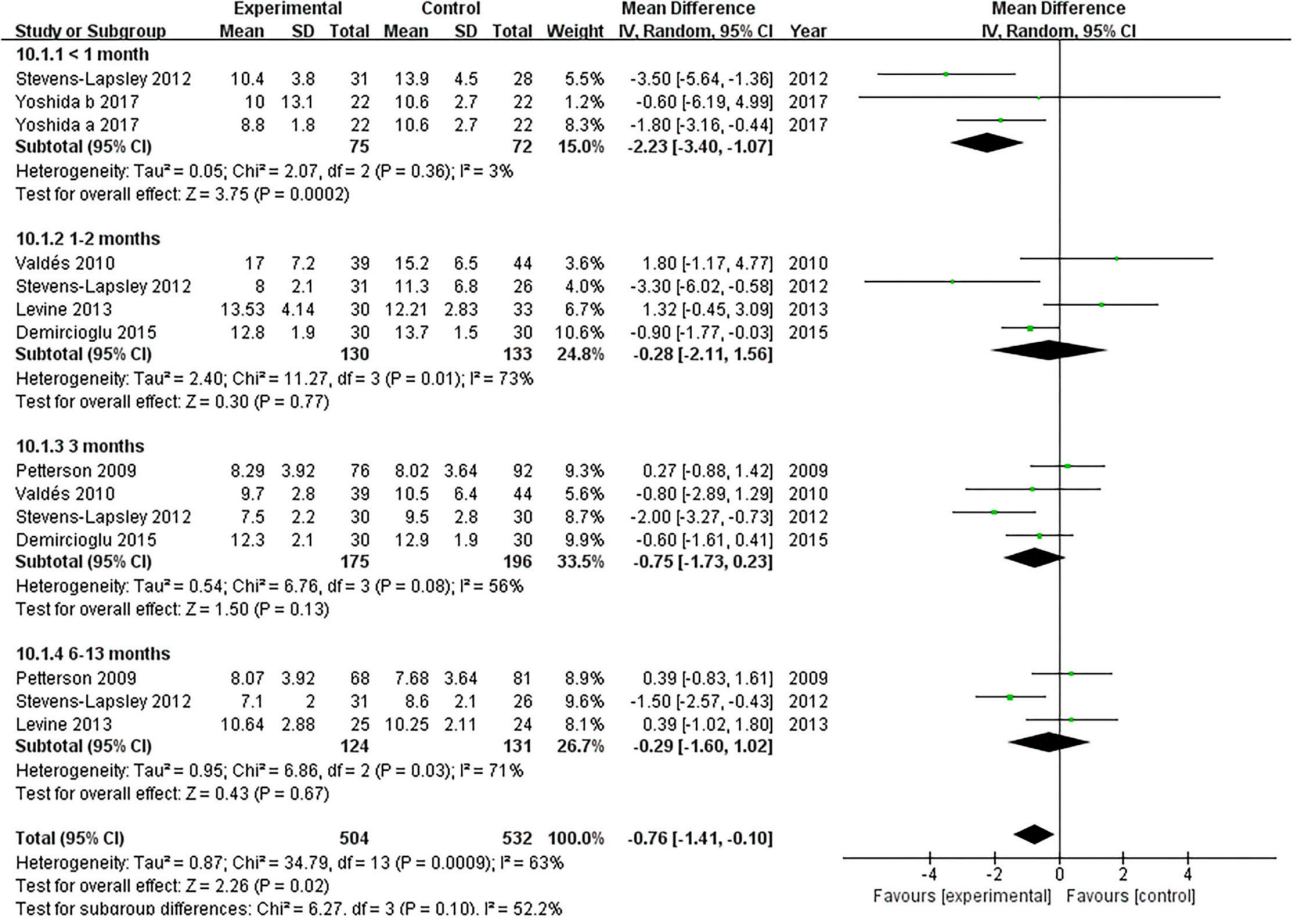
Forest plots of meta-analysis of the effect of NMES vs. conventional rehabilitation on the timed up and go test.

Six articles documented the effect of NMES on TUG following TKA (19, 22, 36–39). NMES improve TUG within 1 month after TKA compared with the control group (MD: −2.23; 95% CI: −3.40 to −1.07, *P* = 0.0002). There was no significant heterogeneity among the articles (*I*^2^ = 3%). However, NMES could not improve postoperative TUG between 1 and 2 months, 3 months, or 6 and 13 months (MD = −0.28, 95% CI: −2.11–1.56, *P* = 0.77, *I*^2^ = 73%; MD = −0.75, 95% CI: −1.73–0.23, *P* = 0.13, *I*^2^ = 56%; MD = −0.29, 95% CI: −1.60–1.02, *P* = 0.67, *I*^2^ = 71%; respectively). The forest plot of TUG was shown in Figure 8.

#### SCT

**Figure 9:**
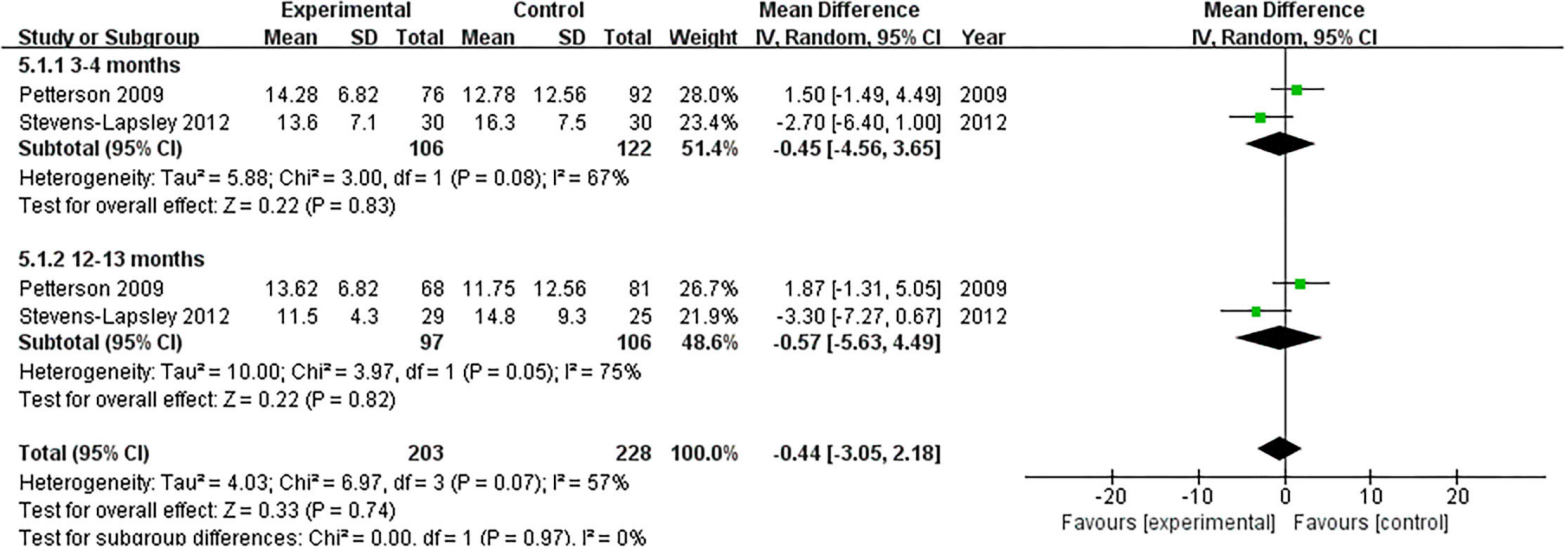
Forest plots of meta-analysis of the effect of NMES vs. conventional rehabilitation on stair-climbing test.

Two RCTs assessed the effect of NMES on SCT following TKA (19, 22). NMES could not improve SCT between 3 and 4 months or 12 and 13 months after TKA (MD: −0.45, 95% CI: −4.56–3.65, *P* = 0.83, *I*^2^ = 67%; MD: −0.57, 95% CI: −5.63–4.49, *P* = 0.82, *I*^2^ = 75%; respectively). The forest plot of SCT was shown in Figure 9.

#### 3MWT

**Figure 10:**
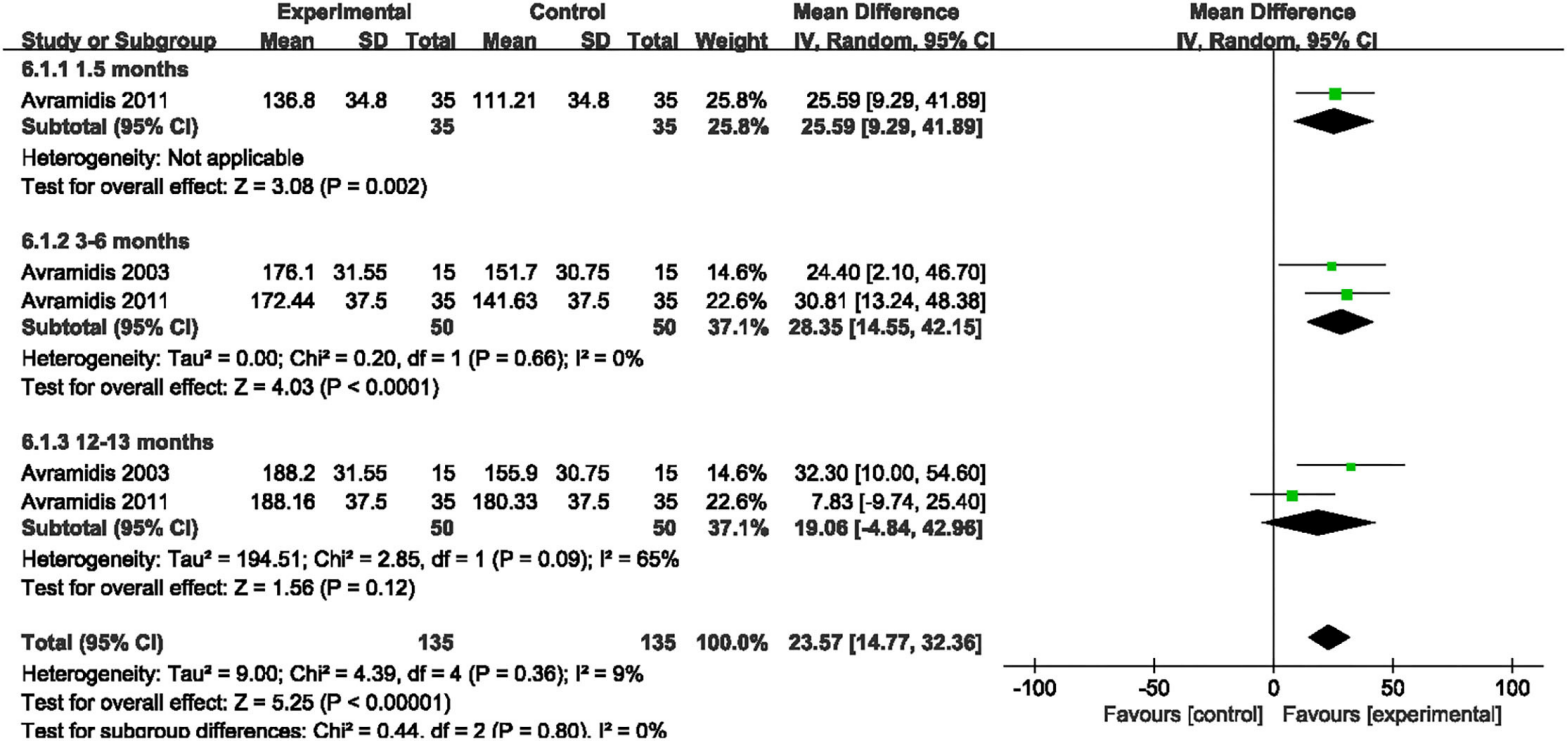
Forest plots of meta-analysis of the effect of NMES vs. conventional rehabilitation on 3 minutes walk test.

Two studies evaluated the effect of NMES on 3MWT following TKA (18, 21). NMES improved 3MWT between 3 and 6 months after TKA compared with the control group (MD: 28.35; 95% CI: 14.55–42.15, *P* < 0.0001). There was no significant heterogeneity among the studies (*I*^2^ = 0%). However, no significant difference was detected on 3MWT between 12 and 13 months after TKA (MD: 19.06; 95% CI: −4.84–42.96, *P* = 0.12, *I*^2^ = 65%). The forest plot of SCT was shown in Figure 10.

#### 6MWT

**Figure 11:**
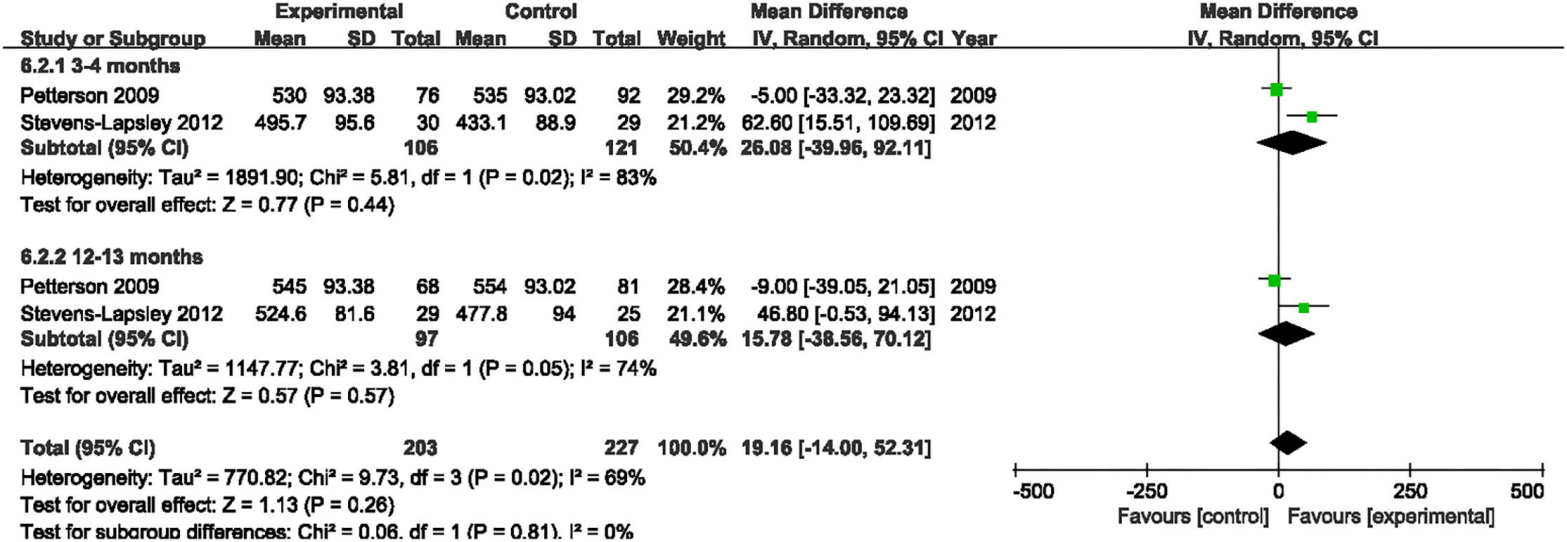
Forest plots of meta-analysis of the effect of NMES vs. conventional rehabilitation on 6-minute walk test.

Two studies evaluated the effect of NMES on 6MWT following TKA (19, 22). Our pooled analysis involving those two studies (19, 22) indicated that NMES could not improve 6MWT between 3 and 4 months or 12 and 13 months after TKA (MD: 26.08, 95% CI: −39.96–92.11, *P* = 0.44, *I*^2^ = 83%; MD: 15.78, 95% CI: −38.56–70.12, *P* = 0.57, *I*^2^ = 74%; respectively). The forest plot of 6MWT was shown in Figure 11.

#### ROM

**Figure 12:**
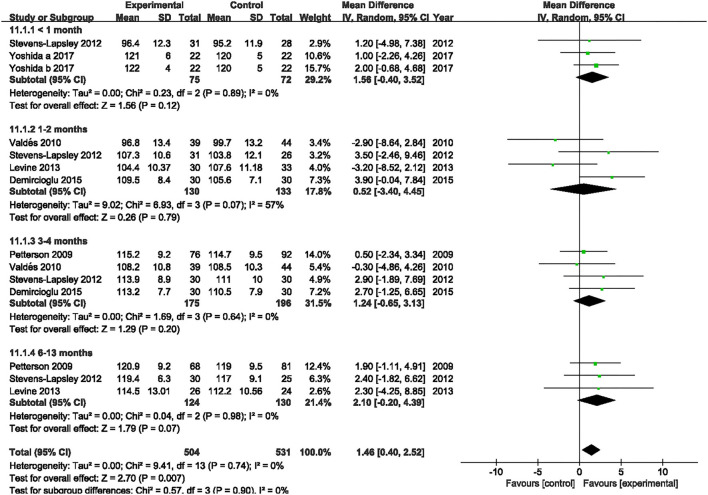
Forest plots of meta-analysis of the effect of NMES vs. conventional rehabilitation on knee flexion.

**Figure 13:**
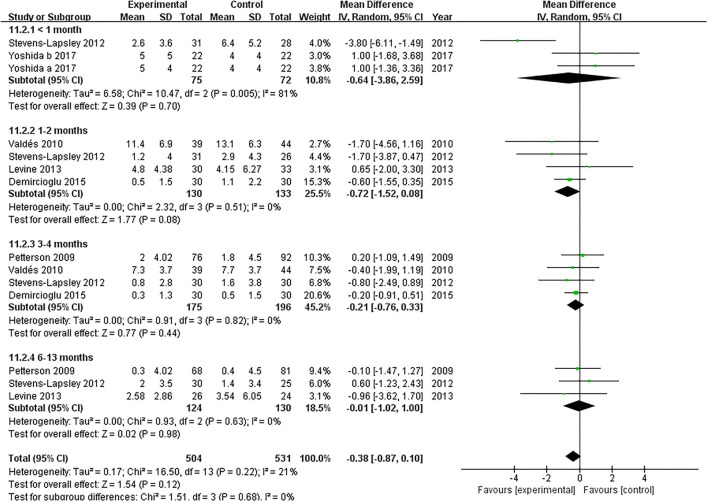
Forest plots of meta-analysis of the effect of NMES vs. conventional rehabilitation on knee extension.

Six studies evaluated the effect of NMES on knee flexion following TKA (19, 22, 36–39). NMES could not improve knee flexion within 1 month, 1 and 2 months, 3 and 4 months, or 6 and 13 months (MD: 1.56, 95% CI: −0.40–3.52, *P* = 0.12, *I*^2^ = 0%; MD = 0.52, 95% CI: −3.40–4.45, *P* = 0.79, *I*^2^ = 57%; MD: 1.24, 95% CI: −0.65–3.13, *P* = 0.20, *I*^2^ = 0%; MD: 2.10 95% CI: −0.20–4.39, *P* = 0.07; respectively). The forest plot of knee flexion was shown in Figure 12. Besides, the same six studies evaluated the effect of NMES on knee extension following TKA (19, 22, 36–39). NMES could not improve knee extension within 1 month, 1–2 months, 3–4 months, or 6–13 months (MD: −0.64, 95% CI: −3.86–2.59, *P* = 0.70, *I*^2^ = 81%; MD = −0.72, 95% CI: −1.52–0.08, *P* = 0.08, *I*^2^ = 0%; MD: −0.21, 95% CI: −0.76–0.33, *P* = 0.44, *I*^2^ = 0%; MD: −0.01, 95% CI: −1.02–1.00, *P* = 0.98, *I*^2^ = 0%; respectively). The forest plot of knee extension was shown in Figure 13.

#### SF-36

**Figure 14:**
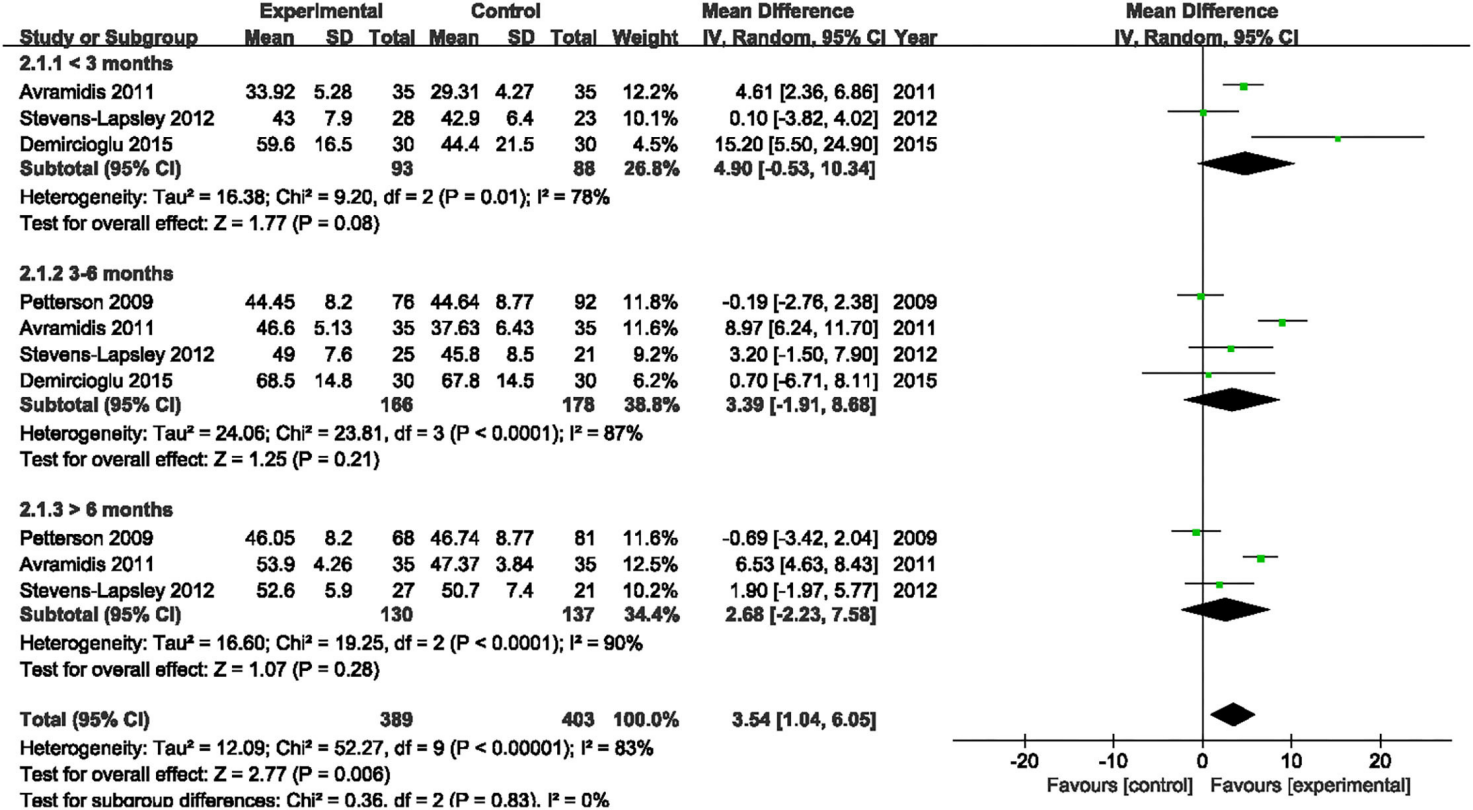
Forest plots of meta-analysis of the effect of NMES vs. conventional rehabilitation on SF-36 PCS.

**Figure 15:**
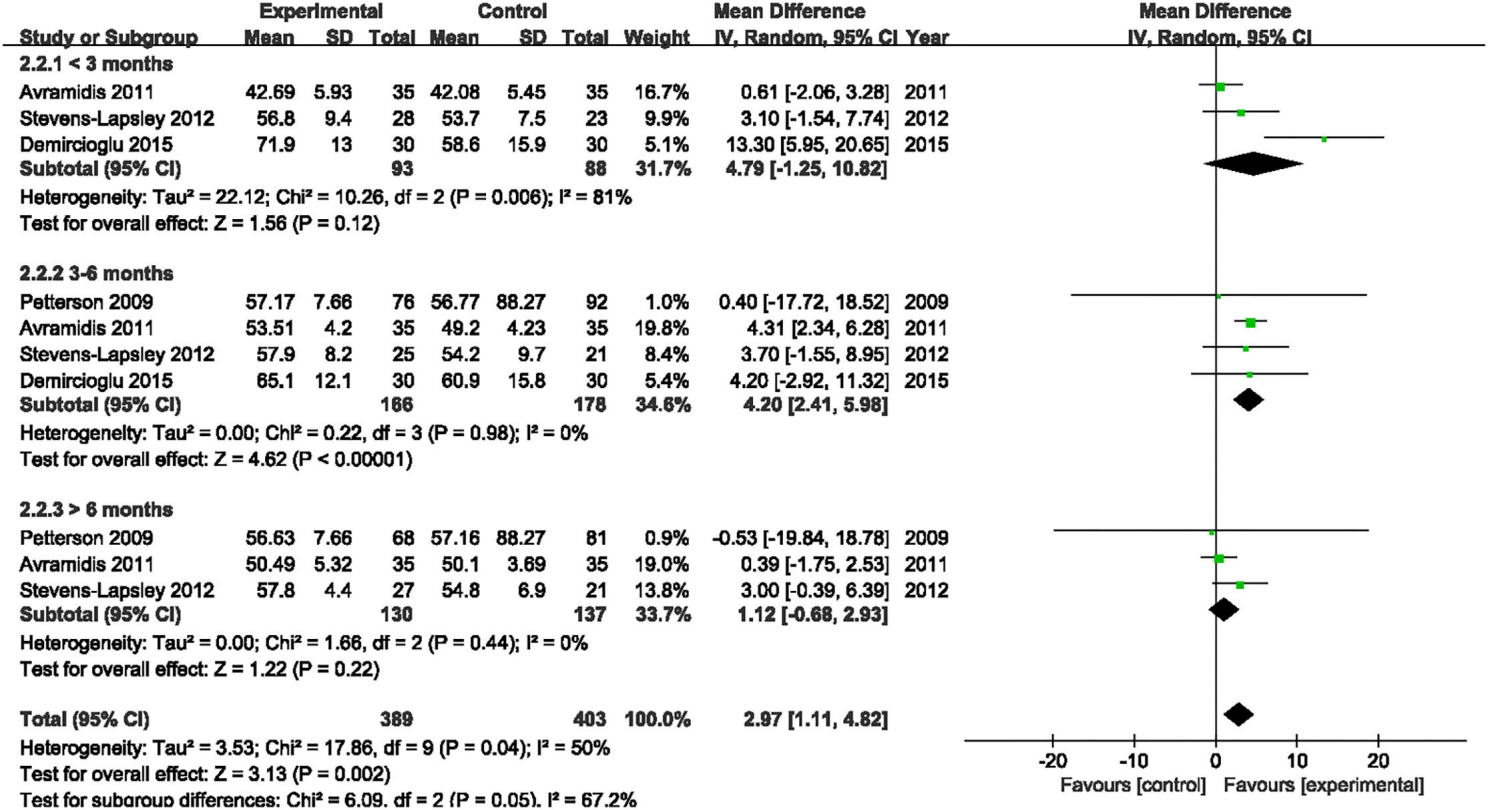
Forest plots of meta-analysis of the effect of NMES vs. conventional rehabilitation on SF-36 MCS.

Four studies assessed the effect of NMES on SF-36 PCS following TKA (19, 21, 22, 38). NMES could not improve SF-36 PCS after TKA within 3 months, 3–6 months, or more than 6 months (MD: 4.90, 95% CI: −0.53–10.34, *P* = 0.08, *I*^2^ = 78%; MD: 3.39, 95% CI: −1.91–8.68, *P* = 0.21, *I*^2^ = 87%; MD = 2.68, 95% CI: −2.23–7.58, *P* = 0.28, *I*^2^ = 90%). The forest plot of SF-36 PCS was shown in Figure 14. The same four studies also assessed the effect of NMES on SF-36 MCS following TKA (19, 21, 22, 38). NMES could not improve SF-36 MCS within 3 months, or more than 6 months (MD: 4.79, 95% CI: −1.25–10.82, *P* = 0.12, *I*^2^ = 81%; MD: 1.12, 95% CI: −0.68–2.93, *P* = 0.22, *I*^2^ = 0%, respectively). However, NMES improved SF-36 MCS between 3 and 6 months after TKA (MD: 4.20, 95% CI: 2.41–5.98, *P* < 0.01). No significant difference was detected (*I*^2^ = 0%). The forest plot of SF-36 MCS was shown in Figure 15.

## Discussion

The purpose of this systematic review and meta-analysis was to explore the effect of NMES on quadriceps muscle strength, pain, and function following TKA. The most important finding of the current study was that postoperative NMES could improve the short-term to long-term quadriceps muscle strength, mid-term pain, and mid-term function following the TKA surgery.

Quadriceps muscle weakness is common following TKA (41). It was reported that 50–60% quadriceps muscle strength deficits might occur compared with the preoperative levels (23). Besides, quadriceps weakness has been found to increase joint loading and contribute to the progress of osteoarthritis (42, 43). It is a crucial goal to restore quadriceps strength for postoperative rehabilitation. Monaghan et al. systematically reviewed relative studies up to 2008. They did not find direct evidence to prove the advantage of NMES on quadriceps muscle strength recovery following TKA by limited (only two) included RCTs (44). Conley et al. conducted a systematic review involving eight RCTs to assess the effect of NMES on quadriceps strength after knee surgery, such as anterior cruciate ligament reconstruction (*n* = 5), TKA (*n* = 2), and meniscectomy (*n* = 1) (43). They revealed that NMES improved the recovery of quadriceps strength after the knee surgery with grade B evidence (43). Due to limited pieces of relevant literature published, some other reviews also failed to examine the effect of NMES on MVIC following TKA (45, 46). After including more RCTs that have been published recently, for the first time, we proved that NMES improved quadriceps muscle strength in terms of MVIC after TKA within 1 month, 1–2 months, 3–4 months, and 12–13 months with high-quality evidence.

After including only one published RCT, one previous review failed to explore the pooled effect of NMES on PCI following TKA (45). We found that NMES could not improve PCI after TKA with high-quality evidence.

There was a high risk of severe acute postoperative pain following TKA, undermining the recovery and delaying the fast-track rehabilitation programs (47). Dabadghav et al. included 28 bilateral TKA patients following osteoarthritis. One knee received NMES plus exercise therapy randomly, and the other knee received exercise merely. After immediate postsurgical rehabilitation of 7 days, no significant difference between the two knees in terms of pain was detected (48). A matched comparison trial demonstrated that patients using the home-based NMES in the first 6 weeks relieved the pain (49). There was no previous meta-analysis that has evaluated the effect of NMES on postoperative pain after TKA. We included five RCTs and found that NMES improved postoperative pain at mid-term (1–2 months and 3–6 months) following TKA. However, the improved differences did not reach the minimal clinically important difference (MCID) in pain (50).

WOMAC is one of the most commonly used questionnaires that assess symptoms and physical function in patients with lower limb osteoarthritis (51, 52). Additional NMES therapy of 8 weeks to exercise could not improve the WOMAC in patients with knee OA (53). No previous meta-analysis pooled analyzed the effect of NMES on postoperative function after TKA. We found that NMES improved postoperative WOMAC slightly at mid-term (3–4 months) following TKA with high-quality evidence.

After a matched comparison trial of 6 weeks, patients who used the home-based NMES improved TUG compared with patients in the control group following TKA (49). No previous meta-analysis analyzed the effect of NMES on postoperative TUG following TKA. With high-quality evidence, we found that NMES improved the postoperative TUG at short-term (within 1 month) following TKA. Bruce-Brand et al. found that patients who received home-based NMES for 6 weeks improved the SCT results than patients who received standard care in knee osteoarthritis (54). We discovered that NMES could not improve postoperative SCT with low-quality evidence. The effect of NMES on early TUG (within 1 month) has not been explored in previous literature. The SCT comprises different movements, such as stair ascending, descending, and their transition (55), which may be hard for patients to conduct in the short-term following TKA.

We found that NMES improved postoperative 3MWT at mid-term with high-quality evidence but not long-term with low-quality evidence. Another two RCTs reported the effect of NMES on 6MWT in our study. We found no significant difference between NMES and control groups in terms of 6MWT at mid-term or long-term. The previous studies emphasized an excellent correlation between 3MWT and 6MWT. 3MWT was easier to learn and repeat than 6MWT for patients (30). The advantage of NMES was found in terms of 3MWT but not 6MWT. The discrepancy may be related to the limited RCTs.

We detected no advantage of NMES in ROM in the present study, such as knee flexion and extension. Dabadghav et al. included 28 postoperative bilateral TKA patients and randomly allocated one knee to NMES plus exercise, while the other knee received exercise (48). They demonstrated no additional effect in terms of ROM between the two knees from 28 bilateral TKA patients (48). Results from an RCT also showed that NMES could not improve the ROM of the hemiplegic shoulder in patients after stroke (56).

We included four RCTs (involving 344 participants) and found that NMES improved SF-36 MCS at mid-term (3–6 months). No differences were found in terms of MCS at short-term or long-term. Besides, NMES could not improve PCS at short-term, mid-term, or long-term. A previous meta-analysis confirmed the advantage of NMES on SF-36 MCS at mid-term (12 weeks) by Bistolfi et al. (45). However, they did not explore the effect of NMES on SF-36 at other periods.

Compared with a previous meta-analysis, Bistolfi et al. only included four RCTs and pooled evaluate the effect of NMES on SF-36 merely (45). The evidence to prove the advantage of NMES in TKA was limited. Given the insufficiency of available data for comparison, some other reviews failed to explore the quantized effect of NMES on TKA (43, 44, 46, 57, 58). As far as we knew, this was the first systematic review and meta-analysis to comprehensively explore the effect of NMES on quadriceps muscle strength, pain, and function following TKA. However, the differences did not reach the MCID in pain (50). Given the included studies adopt different scales in many essential outcomes, the SMD was used to calculate the total effect of quadriceps muscle strength and WOMAC, which may generate issues with heterogeneity.

The study has several limitations. First, all the included studies failed to achieve the performance bias, which contributed to the main bias of this systematic review and meta-analysis. Second, the programs of NMES were not standardized among the included RCTs, which contributes to the heterogeneity. Third, the sample size was relatively small.

## Conclusion

As a supplementary treatment after TKA, postoperative NMES could improve the short-term to long-term quadriceps muscle strength, mid-term pain, and mid-term function following TKA. However, many outcomes failed to achieve statistically meaningful changes and MCID, thus the clinical benefits remained to be confirmed.

## Data Availability Statement

The original contributions presented in the study are included in the article/supplementary material, further inquiries can be directed to the corresponding author.

## Author Contributions

BS and LP conceived and designed the analysis. LP and KW search studies from the databases and analyzed data. YZ, YW, and HS participated in the selection of the studies. LP, YW, and HS extract the data. KW, YZ, and BS participated in the quality assessment. LP drafted the manuscript. BS and YZ ensured the accuracy of the data and analysis. All authors have read and approved the manuscript.

## Funding

This study was supported through grants from the National Natural Science Foundation of China (81974347) and the Clinical Research Incubation project of West China Hospital, Sichuan University (2018HXFH040).

## Conflict of Interest

The authors declare that the research was conducted in the absence of any commercial or financial relationships that could be construed as a potential conflict of interest.

## Publisher's Note

All claims expressed in this article are solely those of the authors and do not necessarily represent those of their affiliated organizations, or those of the publisher, the editors and the reviewers. Any product that may be evaluated in this article, or claim that may be made by its manufacturer, is not guaranteed or endorsed by the publisher.

## Abbreviations

NMES: neuromuscular electrical stimulation
TKA: total knee arthroplasty
PRISMA: preferred reporting items for systematic reviews and meta-analyses
ROM: range of motion
RCT: randomized controlled trial
MCS: mental component score
PCS: physical component score
SF-36: 36-item short form health survey
CENTRAL: Cochrane central register of controlled trials
CNKI: China national knowledge infrastructure
PCI: physiological cost index
VAS: visual analog scale
NPRS: numerical pain rating scale
WOMAC: western ontario and mcmaster universities osteoarthritis index
TUG: timed up and go test
SCT: stair climbing test
3MWT: 3 minutes walk test
6MWT: 6 minutes walk test
MD: mean difference
SMD: standardized mean difference
MVIC: maximal voluntary isometric contraction
BMI: body mass index
mNMES: motor-level NMES
sNMES: sensory-level NMES
KOS ADLS: knee outcome survey activities of daily living scale
HRQoL: health-related quality-of-life
MCID: minimal clinically important difference.
