# Supplementary material for: Effect of Neuromuscular Electrical Stimulation After Total Knee Arthroplasty: A Systematic Review and Meta-Analysis of Randomized Controlled Trials
Source: Front Med (Lausanne). 2021 Dec 3;8:779019. doi: 10.3389/fmed.2021.779019 (PMC8677678; doi:10.3389/fmed.2021.779019)
Supplement: Supplementary file 1 [file Data_Sheet_1.docx]

Search Strategies

Database: PubMed/Medline

Date searched: 18 June 2021

#1 Arthroplasty, Replacement, Knee[MeSH Major Topic]

#2 Arthroplasties, Replacement, Knee OR Arthroplasty, Knee Replacement OR Knee Replacement Arthroplasties OR Knee Replacement Arthroplasty OR Replacement Arthroplasties, Knee OR Knee Arthroplasty, Total OR Arthroplasty, Total Knee OR Total Knee Arthroplasty OR Replacement, Total Knee OR Total Knee Replacement OR Knee Replacement, Total OR Knee Arthroplasty OR Arthroplasty, Knee OR Arthroplasties, Knee Replacement OR Replacement Arthroplasty, Knee

#3 #1 OR #2

#4 Electric Stimulation[MeSH Major Topic]

#5 Electric Stimulation Therapy[MeSH Major Topic]

#6 (Electrical AND Stimulation) OR (Electrical AND Stimulations) OR (Stimulation, Electrical) OR (Stimulations, Electrical) OR (Stimulation, Electric) OR (Electric AND Stimulations) OR (Stimulations, Electric) OR (Therapeutic AND Electrical AND Stimulation) OR (Electrical AND Stimulation, Therapeutic) OR (Stimulation, Therapeutic AND Electrical) OR (Therapeutic AND Electric AND Stimulation) OR (Electric AND Stimulation, Therapeutic) OR (Stimulation, Therapeutic AND Electric) OR (Electrical AND Stimulation AND Therapy) OR (Stimulation Therapy, Electrical) OR (Therapy, Electrical AND Stimulation) OR (Therapy, Electric AND Stimulation) OR (Stimulation AND Therapy, Electric) OR Electrotherapy OR (Interferential AND Current AND Electrotherapy) OR (Electrotherapy, Interferential AND Current) OR EMS OR NMES

#7 #4 OR #5 OR #6

#8 #3 AND #7

#9 ((((((randomized controlled trial [pt]) OR (controlled clinical trial [pt])) OR (randomized [tiab])) OR (placebo [tiab])) OR (clinical trials as topic [mesh: noexp])) OR (randomly [tiab])) OR (trial [ti])

#10 animals [mh] NOT humans [mh]

#11 #9 NOT #10

#12 #8 AND #11

Database: Embase

Date searched: 18 June 2021

#1 'total knee arthroplasty'/exp

#2 'knee arthroplasty, total':ab,ti

#3 'knee replacement, total':ab,ti

#4 'total knee joint replacement':ab,ti

#5 'total knee replacement':ab,ti

#6 'total knee replacement arthroplasty':ab,ti

#7 #1 OR #2 OR #3 OR #4 OR #5 OR #6

#8 'electrostimulation'/exp

#9 'electrotherapy'/exp

#10 'neuromuscular electrical stimulation'/exp

#11 'Electrical AND Stimulation':ab,ti OR 'Electrical AND Stimulations':ab,ti OR 'Stimulation, Electrical':ab,ti OR 'Stimulations, Electrical':ab,ti OR 'Stimulation, Electric':ab,ti OR 'Electric AND Stimulations':ab,ti OR 'Stimulations, Electric':ab,ti OR 'Therapeutic AND Electrical AND Stimulation':ab,ti OR 'Electrical AND Stimulation, Therapeutic':ab,ti OR 'Stimulation, Therapeutic AND Electrical':ab,ti OR 'Therapeutic AND Electric AND Stimulation':ab,ti OR 'Electric AND Stimulation, Therapeutic':ab,ti OR 'Stimulation, Therapeutic AND Electric':ab,ti OR 'Electrical AND Stimulation AND Therapy':ab,ti OR 'Stimulation Therapy, Electrical':ab,ti OR 'Therapy, Electrical AND Stimulation':ab,ti OR 'Therapy, Electric AND Stimulation':ab,ti OR 'Stimulation AND Therapy, Electric':ab,ti OR 'Electrotherapy':ab,ti OR 'Interferential AND Current AND Electrotherapy':ab,ti OR 'Electrotherapy, Interferential AND Current':ab,ti OR 'EMS':ab,ti OR 'NMES':ab,ti

#12 #8 OR #9 OR #10 OR #11

#13 'randomized controlled trial'/exp

#14 #7 AND #12 AND #13

Database: Web of Science

Date searched: 18 June 2021

#1

TI=(Arthroplasty, Replacement, Knee) OR AB=(Arthroplasty, Replacement, Knee) OR TI=(Arthroplasties, Replacement, Knee OR Arthroplasty, Knee Replacement OR Knee Replacement Arthroplasties OR Knee Replacement Arthroplasty OR Replacement Arthroplasties, Knee OR Knee Arthroplasty, Total OR Arthroplasty, Total Knee OR Total Knee Arthroplasty OR Replacement, Total Knee OR Total Knee Replacement OR Knee Replacement, Total OR Knee Arthroplasty OR Arthroplasty, Knee OR Arthroplasties, Knee Replacement OR Replacement Arthroplasty, Knee) OR AB=(Arthroplasties, Replacement, Knee OR Arthroplasty, Knee Replacement OR Knee Replacement Arthroplasties OR Knee Replacement Arthroplasty OR Replacement Arthroplasties, Knee OR Knee Arthroplasty, Total OR Arthroplasty, Total Knee OR Total Knee Arthroplasty OR Replacement, Total Knee OR Total Knee Replacement OR Knee Replacement, Total OR Knee Arthroplasty OR Arthroplasty, Knee OR Arthroplasties, Knee Replacement OR Replacement Arthroplasty, Knee)

#2

TI=((Electrical AND Stimulation) OR (Electrical AND Stimulations) OR (Stimulation, Electrical) OR (Stimulations, Electrical) OR (Stimulation, Electric) OR (Electric AND Stimulations) OR (Stimulations, Electric) OR (Therapeutic AND Electrical AND Stimulation) OR (Electrical AND Stimulation, Therapeutic) OR (Stimulation, Therapeutic AND Electrical) OR (Therapeutic AND Electric AND Stimulation) OR (Electric AND Stimulation, Therapeutic) OR (Stimulation, Therapeutic AND Electric) OR (Electrical AND Stimulation AND Therapy) OR (Stimulation Therapy, Electrical) OR (Therapy, Electrical AND Stimulation) OR (Therapy, Electric AND Stimulation) OR (Stimulation AND Therapy, Electric) OR Electrotherapy OR (Interferential AND Current AND Electrotherapy) OR (Electrotherapy, Interferential AND Current) OR EMS OR NMES OR (Neuromuscular AND Electrical AND Stimulation)) OR AB=((Electrical AND Stimulation) OR (Electrical AND Stimulations) OR (Stimulation, Electrical) OR (Stimulations, Electrical) OR (Stimulation, Electric) OR (Electric AND Stimulations) OR (Stimulations, Electric) OR (Therapeutic AND Electrical AND Stimulation) OR (Electrical AND Stimulation, Therapeutic) OR (Stimulation, Therapeutic AND Electrical) OR (Therapeutic AND Electric AND Stimulation) OR (Electric AND Stimulation, Therapeutic) OR (Stimulation, Therapeutic AND Electric) OR (Electrical AND Stimulation AND Therapy) OR (Stimulation Therapy, Electrical) OR (Therapy, Electrical AND Stimulation) OR (Therapy, Electric AND Stimulation) OR (Stimulation AND Therapy, Electric) OR Electrotherapy OR (Interferential AND Current AND Electrotherapy) OR (Electrotherapy, Interferential AND Current) OR EMS OR NMES OR (Neuromuscular AND Electrical AND Stimulation))

#3 #1 AND #2

Database: the Cochrane Library

Date searched: 18 June 2021

#1 MeSH descriptor: [Arthroplasty, Replacement, Knee] explode all trees

#2 MeSH descriptor: [Electric Stimulation] explode all trees

#3 MeSH descriptor: [Electric Stimulation Therapy] explode all trees

#4 (Arthroplasties, Replacement, Knee) OR (Arthroplasty, Knee Replacement) OR (Knee Replacement Arthroplasties) OR (Knee Replacement Arthroplasty) OR (Replacement Arthroplasties, Knee) OR (Knee Arthroplasty, Total) OR (Arthroplasty, Total Knee) OR (Total Knee Arthroplasty) OR (Replacement, Total Knee) OR (Total Knee Replacement) OR (Knee Replacement, Total) OR (Knee Arthroplasty) OR (Arthroplasty, Knee) OR (Arthroplasties, Knee Replacement) OR (Replacement Arthroplasty, Knee):ti,ab,kw (Word variations have been searched)

#5 (Electrical AND Stimulation) OR (Electrical AND Stimulations) OR (Stimulation, Electrical) OR (Stimulations, Electrical) OR (Stimulation, Electric) OR (Electric AND Stimulations) OR (Stimulations, Electric) OR (Therapeutic AND Electrical AND Stimulation) OR (Electrical AND Stimulation, Therapeutic) OR (Stimulation, Therapeutic AND Electrical) OR (Therapeutic AND Electric AND Stimulation) OR (Electric AND Stimulation, Therapeutic) OR (Stimulation, Therapeutic AND Electric) OR (Electrical AND Stimulation AND Therapy) OR (Stimulation Therapy, Electrical) OR (Therapy, Electrical AND Stimulation) OR (Therapy, Electric AND Stimulation) OR (Stimulation AND Therapy, Electric) OR Electrotherapy OR (Interferential AND Current AND Electrotherapy) OR (Electrotherapy, Interferential AND Current) OR EMS OR NMES

#6 #1 OR #4

#7 #2 OR #3 OR #5

#8 #6 AND #7

Proquest

Date searched: 18 June 2021

1# Arthroplasty, Replacement, Knee OR Arthroplasties, Replacement, Knee OR Arthroplasty, Knee Replacement OR Knee Replacement Arthroplasties OR Knee Replacement Arthroplasty OR Replacement Arthroplasties, Knee OR Knee Arthroplasty, Total OR Arthroplasty, Total Knee OR Total Knee Arthroplasty OR Replacement, Total Knee OR Total Knee Replacement OR Knee Replacement, Total OR Knee Arthroplasty OR Arthroplasty, Knee OR Arthroplasties, Knee Replacement OR Replacement Arthroplasty, Knee

(Arthroplasty, Replacement, Knee) OR (Arthroplasties, Replacement, Knee) OR (Arthroplasty, Knee Replacement) OR (Knee Replacement Arthroplasties) OR (Knee Replacement Arthroplasty) OR (Replacement Arthroplasties, Knee) OR (Knee Arthroplasty, Total) OR (Arthroplasty, Total Knee) OR (Total Knee Arthroplasty) OR (Replacement, Total Knee) OR (Total Knee Replacement) OR (Knee Replacement, Total) OR (Knee Arthroplasty) OR (Arthroplasty, Knee) OR (Arthroplasties, Knee Replacement) OR (Replacement Arthroplasty, Knee)

2# (Electrical AND Stimulation) OR (Electrical AND Stimulations) OR (Stimulation, Electrical) OR (Stimulations, Electrical) OR (Stimulation, Electric) OR (Electric AND Stimulations) OR (Stimulations, Electric) OR (Therapeutic AND Electrical AND Stimulation) OR (Electrical AND Stimulation, Therapeutic) OR (Stimulation, Therapeutic AND Electrical) OR (Therapeutic AND Electric AND Stimulation) OR (Electric AND Stimulation, Therapeutic) OR (Stimulation, Therapeutic AND Electric) OR (Electrical AND Stimulation AND Therapy) OR (Stimulation Therapy, Electrical) OR (Therapy, Electrical AND Stimulation) OR (Therapy, Electric AND Stimulation) OR (Stimulation AND Therapy, Electric) OR Electrotherapy OR (Interferential AND Current AND Electrotherapy) OR (Electrotherapy, Interferential AND Current) OR EMS OR NMES OR (Neuromuscular AND Electrical AND Stimulation)

1#2

**Scopus**

Date searched: 18 June 2021

TITLE-ABS-KEY((Arthroplasty, Replacement, Knee) OR (Arthroplasties, Replacement, Knee) OR (Arthroplasty, Knee Replacement) OR (Knee Replacement Arthroplasties) OR (Knee Replacement Arthroplasty) OR (Replacement Arthroplasties, Knee) OR (Knee Arthroplasty, Total) OR (Arthroplasty, Total Knee) OR (Total Knee Arthroplasty) OR (Replacement, Total Knee) OR (Total Knee Replacement) OR (Knee Replacement, Total) OR (Knee Arthroplasty) OR (Arthroplasty, Knee) OR (Arthroplasties, Knee Replacement) OR (Replacement Arthroplasty, Knee)) AND TITLE-ABS-KEY((Electrical AND Stimulation) OR (Electrical AND Stimulations) OR (Stimulation, Electrical) OR (Stimulations, Electrical) OR (Stimulation, Electric) OR (Electric AND Stimulations) OR (Stimulations, Electric) OR (Therapeutic AND Electrical AND Stimulation) OR (Electrical AND Stimulation, Therapeutic) OR (Stimulation, Therapeutic AND Electrical) OR (Therapeutic AND Electric AND Stimulation) OR (Electric AND Stimulation, Therapeutic) OR (Stimulation, Therapeutic AND Electric) OR (Electrical AND Stimulation AND Therapy) OR (Stimulation Therapy, Electrical) OR (Therapy, Electrical AND Stimulation) OR (Therapy, Electric AND Stimulation) OR (Stimulation AND Therapy, Electric) OR Electrotherapy OR (Interferential AND Current AND Electrotherapy) OR (Electrotherapy, Interferential AND Current) OR EMS OR NMES OR (Neuromuscular AND Electrical AND Stimulation)) AND TITLE-ABS-KEY((randomized controlled trial*) OR (controlled clinical trial*) OR (randomized) OR (placebo*) OR (clinical trial*) OR (random*) OR (trial*))

CINAHL

Date searched: 18 June 2021

#S1
TI ( (Arthroplasty, Replacement, Knee) OR (Arthroplasties, Replacement, Knee) OR (Arthroplasty, Knee Replacement) OR (Knee Replacement Arthroplasties) OR (Knee Replacement Arthroplasty) OR (Replacement Arthroplasties, Knee) OR (Knee Arthroplasty, Total) OR (Arthroplasty, Total Knee) OR (Total Knee Arthroplasty) OR (Replacement, Total Knee) OR (Total Knee Replacement) OR (Knee Replacement, Total) OR (Knee Arthroplasty) OR (Arthroplasty, Knee) OR (Arthroplasties, Knee Replacement) OR (Replacement Arthroplasty, Knee) ) OR AB ( (Arthroplasty, Replacement, Knee) OR (Arthroplasties, Replacement, Knee) OR (Arthroplasty, Knee Replacement) OR (Knee Replacement Arthroplasties) OR (Knee Replacement Arthroplasty) OR (Replacement Arthroplasties, Knee) OR (Knee Arthroplasty, Total) OR (Arthroplasty, Total Knee) OR (Total Knee Arthroplasty) OR (Replacement, Total Knee) OR (Total Knee Replacement) OR (Knee Replacement, Total) OR (Knee Arthroplasty) OR (Arthroplasty, Knee) OR (Arthroplasties, Knee Replacement) OR (Replacement Arthroplasty, Knee) )

#S2
TI ( (Electrical AND Stimulation) OR (Electrical AND Stimulations) OR (Stimulation, Electrical) OR (Stimulations, Electrical) OR (Stimulation, Electric) OR (Electric AND Stimulations) OR (Stimulations, Electric) OR (Therapeutic AND Electrical AND Stimulation) OR (Electrical AND Stimulation, Therapeutic) OR (Stimulation, Therapeutic AND Electrical) OR (Therapeutic AND Electric AND Stimulation) OR (Electric AND Stimulation, Therapeutic) OR (Stimulation, Therapeutic AND Electric) OR (Electrical AND Stimulation AND Therapy) OR (Stimulation Therapy, Electrical) OR (Therapy, Electrical AND Stimulation) OR (Therapy, Electric AND Stimulation) OR (Stimulation AND Therapy, Electric) OR Electrotherapy OR (Interferential AND Current AND Electrotherapy) OR (Electrotherapy, Interferential AND Current) OR EMS OR NMES OR (Neuromuscular AND Electrical AND Stimulation) ) OR AB ( (Electrical AND Stimulation) OR (Electrical AND Stimulations) OR (Stimulation, Electrical) OR (Stimulations, Electrical) OR (Stimulation, Electric) OR (Electric AND Stimulations) OR (Stimulations, Electric) OR (Therapeutic AND Electrical AND Stimulation) OR (Electrical AND Stimulation, Therapeutic) OR (Stimulation, Therapeutic AND Electrical) OR (Therapeutic AND Electric AND Stimulation) OR (Electric AND Stimulation, Therapeutic) OR (Stimulation, Therapeutic AND Electric) OR (Electrical AND Stimulation AND Therapy) OR (Stimulation Therapy, Electrical) OR (Therapy, Electrical AND Stimulation) OR (Therapy, Electric AND Stimulation) OR (Stimulation AND Therapy, Electric) OR Electrotherapy OR (Interferential AND Current AND Electrotherapy) OR (Electrotherapy, Interferential AND Current) OR EMS OR NMES OR (Neuromuscular AND Electrical AND Stimulation) )

#S3 S1 AND S2

PEDro

Date searched: 18 June 2021

TI OR AB (total knee arthroplasty OR total knee replacement)

THERAPY (electrotherapies)

Method (Clinical trial)

Database: CNKI

Date searched: 18 June 2021

1 SU= (SU='全膝关节置换术' OR SU='全膝关节置换' OR SU='膝关节置换' OR TI='全膝关节置换术' OR SU='全膝关节置换' OR SU='膝关节置换' OR AB='全膝关节置换术' OR SU='全膝关节置换' OR SU='膝关节置换')

2 (SU='电疗' OR SU='神经肌肉电刺激' OR SU='电刺激' OR TI='电疗' OR TI='神经肌肉电刺激' OR TI='电刺激' OR AB='电疗' OR AB='神经肌肉电刺激' OR AB='电刺激')

3 1 and 2

Database: Wanfang

Date searched: 18 June 2021

1 ( "电疗"[常用字段:智能] OR "神经肌肉电刺激"[常用字段:智能] OR "电刺激"[常用字段:智能])

2 (( "全膝关节置换术"[常用字段:智能] OR "全膝关节置换"[常用字段:智能] OR 膝关节置换"[常用字段:智能]))

3 1 and 2
